# Key Roles of AXL and MER Receptor Tyrosine Kinases in Resistance to Multiple Anticancer Therapies

**DOI:** 10.1007/s11912-017-0579-4

**Published:** 2017-03-01

**Authors:** Marie Schoumacher, Mike Burbridge

**Affiliations:** 1 Oncology Translational and Clinical Research, Institut de Recherches Servier (IdRS), 125 Chemin de Ronde, 78290 Croissy, France; 2 Oncology Translational and Clinical Research, Institut de Recherches Internationales Servier (IRIS), 50 rue Carnot, 92284 Suresnes cedex, France

**Keywords:** AXL, MER, Cancer, Drug resistance, Epithelial-to-mesenchymal transition, Immunomodulation

## Abstract

A major challenge in anticancer treatment is the pre-existence or emergence of resistance to therapy. AXL and MER are two members of the TAM (TYRO3-AXL-MER) family of receptor tyrosine kinases, which, when activated, can regulate tumor cell survival, proliferation, migration and invasion, angiogenesis, and tumor-host interactions. An increasing body of evidence strongly suggests that these receptors play major roles in resistance to targeted therapies and conventional cytotoxic agents. Multiple resistance mechanisms exist, including the direct and indirect crosstalk of AXL and MER with other receptors and the activation of feedback loops regulating AXL and MER expression and activity. These mechanisms may be innate, adaptive, or acquired. A principal role of AXL appears to be in sustaining a mesenchymal phenotype, itself a major mechanism of resistance to diverse anticancer therapies. Both AXL and MER play a role in the repression of the innate immune response which may also limit response to treatment. Small molecule and antibody inhibitors of AXL and MER have recently been described, and some of these have already entered clinical trials. The optimal design of treatment strategies to maximize the clinical benefit of these AXL and MER targeting agents are discussed in relation to the different cancer types and the types of resistance encountered. One of the major challenges to successful development of these therapies will be the application of robust predictive biomarkers for clear-cut patient stratification.

## Introduction

Receptor tyrosine kinases (RTKs) are broadly involved in cellular signaling. Many RTKs are deregulated in cancer, many of them being oncogenic drivers. The TAM family of RTKs comprises three transmembrane receptors: TYRO-3, AXL, and MER. Their extracellular domain resembles to some extent that of cell adhesion molecules and contains two immunoglobulin-like and two fibronectin type III domains, while the intracellular kinase domain mediates activation of signaling pathways. Activation of the receptors is triggered by homodimerization following ligand binding, or ligand-independent mechanisms, such as heterodimerization with other TAM or non-TAM RTKs. Several ligands have been identified, with different affinities towards the three TAM receptors: GAS6, protein S, Tubby, Tubby-like protein 1 (TULP-1), and Galectin-3. More data are available on GAS6 and protein S since they were the first to be identified. GAS6 can bind all three receptors, whereas protein S is specific for MER and TYRO-3. The affinity of GAS6 is, however, 3- to 10-fold higher for AXL compared to MER and TYRO-3.

In normal adult tissues, TAM receptors have widespread expression patterns, being expressed in the brain (hippocampus, cerebellum), heart, and liver as well as in monocytes, platelets, and endothelial cells. Their physiological function resides mostly in the regulation of inflammation and elimination of debris via phagocytosis [1••, 2]. Overall, MER is more specifically expressed by cells from the hematopoietic lineage (monocytes, macrophages, dendritic cells, natural killer cells, platelets) while AXL expression pattern is more constrained to epithelial tissues.

TAM receptors are implicated in the regulation of the innate immune response as well as in several signaling cascades that are essential during cancer progression. Being initially discovered in cancers, their biological functions have mostly been studied in the oncology field [3, 4]. Overexpression of AXL and to a lesser extent of MER has been described in multiple malignancies from epithelial and hematological origins and is often associated with poor prognosis [1••, 5–9]. Several studies highlight the role of AXL activation in tumor progression and metastasis development. In most settings, AXL/MER expression is induced together with the appearance of drug resistance to conventional or targeted therapies (Table 1). Moreover, AXL expression is associated with epithelial to mesenchymal transition (EMT), a frequent feature of metastatic tumors often correlated to drug resistance. Both tumor and stromal cells from the microenvironment can produce GAS6, fostering a crosstalk between the two cell populations. Targeting AXL could thus be a good strategy to overcome drug resistance in multiple cancer types by targeting both tumor cells (mostly via AXL) and their microenvironment (via AXL and MER).

**Table 1:** Resistance to conventional and targeted therapies involving AXL and MER

Resistance setting	Therapy	Cancer type	References
AXL-mediated resistance to targeted therapies	HER2 inhibition	Breast	[35, 58••]
	RAF inhibition	Melanoma	[84, 121–123, 124]
	MEK inhibition	Breast, melanoma	[63•]
	EGFR inhibition	NSCLC	[60, 82•, 115, 125]
	EGFR inhibition	HNSCC	[28]
	Sunitinib	Renal	[117]
	Imatinib	CML	[15]
	Imatinib	GIST	[39]
	ALK inhibition	Neuroblastoma	[126, 127]
	VEGFR inhibition	Diverse	[115, 128]
	PI3K/AKT inhibition	Diverse	[61••, 129]
	FTL-3 inhibition	AML	[130]
AXL-mediated resistance to conventional therapies		AML	[36, 43]
		CML	[131]
		Breast	[88, 131]
		Esophageal	[132]
		Lung	[133]
		Ovarian	[134]
		Colon	[135]
MER-mediated resistance to conventional therapies		B-ALL, T-ALL	[136, 137]
		Glioma	[68]
		Lung	[133, 138]
AXL/MER-mediated resistance to immune checkpoint therapies		Breast	[97•, 98•]
		Colon	[99•]

This review will focus on the role of AXL and MER in drug resistance and how TAM inhibitors could be best used to reverse innate or acquired resistance.

## Regulation of AXL and MER Expression

Although the mechanisms involved are not fully understood, the roles of AXL and MER in cancer are governed essentially by deregulation of transcription leading, directly or indirectly, to increased levels of the activated proteins [1••, 2, 10]. In the few cases where chromosome amplifications, mutations or gene fusions have been described, functional evidence is lacking [11–14]. Mechanisms involved in AXL and MER transcriptional regulation are described below.

### Transcription Factors and Regulators

Five transcription factor complexes have been shown to regulate AXL promoter activity: activator protein 1 (AP1), SP1/SP3, YAP/TAZ/TEAD, hypoxia inducible factor (HIF), and myeloid zinc finger 1 protein (MZF-1). Binding sites for the FOS and JUN components of AP1 are present in the AXL promoter and functional studies have confirmed their involvement in the regulation of AXL expression in chronic myeloid leukemia (CML) and bladder cancer, respectively [15, 16]. The transcription factors SP1 and SP3 bind to GC-rich regions on the AXL promoter to induce its expression. Methylation of CpG sites in these SP binding regions inhibits SP-driven transcription of AXL [17]. The YAP/TAZ/TEAD complex was shown to regulate AXL expression in gallbladder, hepatocellular carcinoma, and lung cancers [18–20]. In particular, one study shows that upregulation of the Hippo/YAP pathway in non-small cell lung cancer (NSCLC) cells induces AXL expression and leads to increased resistance to inhibition of the epidermal growth factor receptor (EGFR) [19]. Interestingly, an association between hypoxia and AXL has been suggested in clear cell renal carcinoma, where hypoxia-responsive elements are present in the proximal region of the AXL promoter and binding of HIF-1 and HIF-2 directly induces AXL expression [21]. Moreover, a correlation between HIF-1 and AXL expression has been shown in metastatic prostate cancer, where hypoxia stabilized the AXL protein [22]. Lastly, one study demonstrates the role of the myeloid zinc finger 1 protein (MZF-1) in colorectal and cervical cancer cell lines [23].

Some studies have highlighted the regulation of *AXL* transcription in cancer through feedback loops induced by other RTKs. In NSCLC and head and neck squamous cell carcinoma (HNSCC) for example, EGFR signaling and downstream MEK/ERK activation induces expression of *AXL* mRNA via the JUN transcription factor [24]. Similar findings have been described in bladder cancer where *AXL* mRNA is induced after MET activation and downstream MEK/ERK signaling [25].

### Alternative Transcriptional Control

Two microRNAs (miRNAs) have been described as repressors of AXL expression: miR-34a and miR-199a/b. These miRNAs bind to the 3′-UTR of the *AXL* gene to negatively regulate its expression in breast, colorectal, head and neck, hepatocellular carcinoma, and lung cancer cell lines [26–31]. Recently, one elegant study showed that the miRNA-processing enzyme Dicer suppresses AXL expression in breast cancer cells by inducing expression of miR-494. As a consequence, cells lose their stem cell-like properties and have increased sensitivity to paclitaxel [32•].

*AXL* gene expression is also governed by epigenetic changes in histone acetylation and histone/DNA methylation. Histone demethylation by EZH2 increases *AXL* mRNA expression in glioma [33]. DNA methylation of *AXL* was detected in NSCLC cell lines and was associated with EMT features and resistance to EGFR inhibition [34]. Promoter hypomethylation is associated with increased expression of AXL in HER2 inhibitor-resistant breast cancers [35], acute myeloid leukemia (AML) [36], and some colorectal models [17]. Histone deacetylase (HDAC) inhibition has been shown to reduce AXL expression in AML, suggesting a link between histone acetylation and AXL expression [37]. One study performed in lung cancer cells suggests that mutant p53 could mediate histone acetylation on the *AXL* promoter, increasing AXL expression and triggering cell growth and motility [38]. A more detailed epigenetic map across tumor types and characterization of the methylation/acetylation status of the *AXL* gene is required to confirm these findings.

## AXL and MER in Resistance Mediated by Feedback Loops and Receptor Crosstalk

### Regulation of AXL and MER Activity

**Fig. 1:**
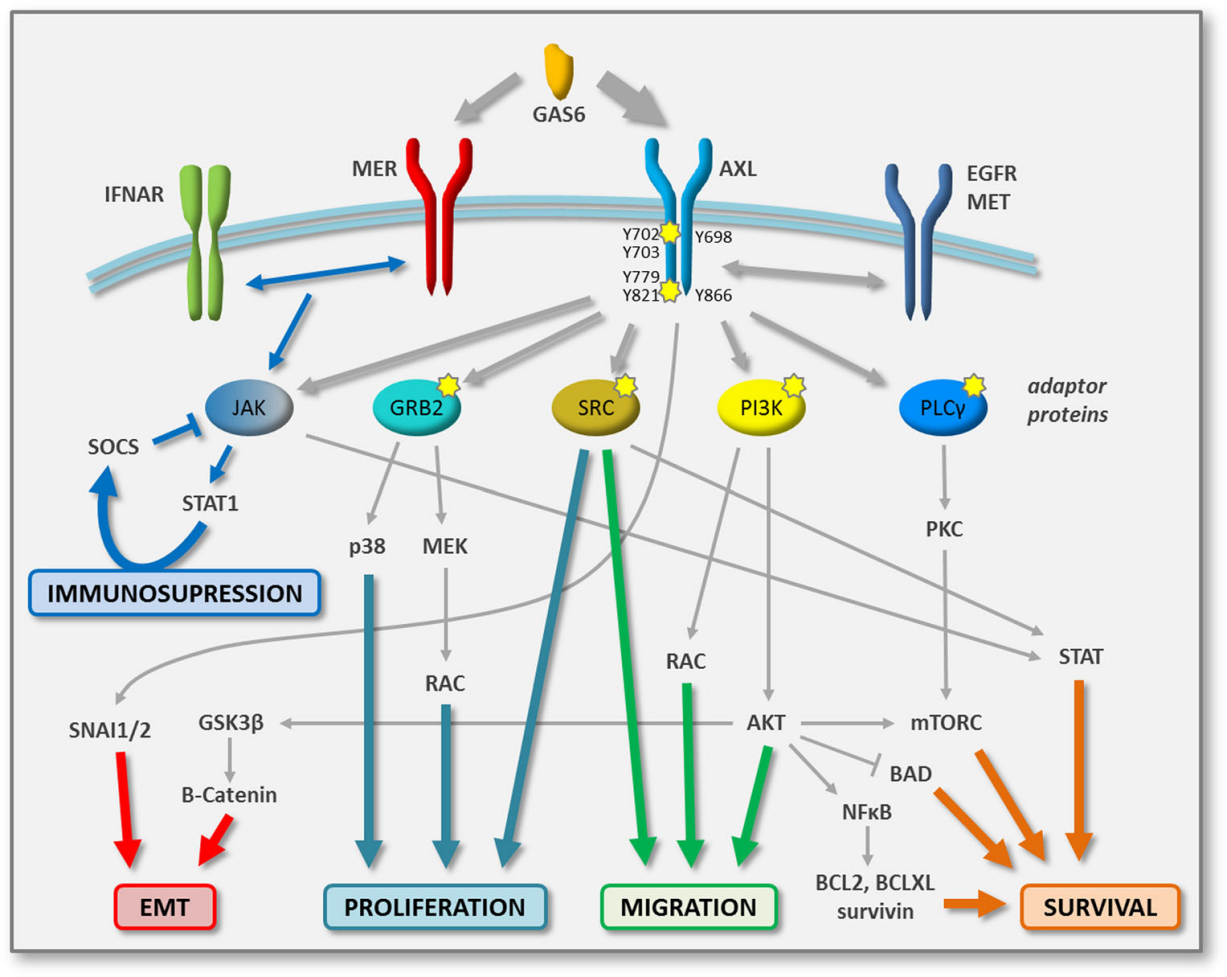
AXL and MER signaling networks in tumor cells. Schematic representing the major signaling networks activated upon binding of GAS6 with its TAM receptor in tumor cells. Affinity of GAS6 for AXL is higher than that for MER. Tyrosine docking sites in AXL are represented. Diverse adaptor proteins mediate activation of specific signaling pathways, involved in proliferation, migration, and survival. Potential direct and indirect phosphorylation biomarkers of AXL activity (pharmacodynamic markers) are indicated by *yellow stars*. Crosstalk between AXL and other RTKs is exemplified by the dimerization with EGFR and MET. Signaling implicated in the regulation of the immune response is depicted by *blue arrows*

Both paracrine and autocrine loops can activate AXL/MER signaling cascades (Fig. 1). Multiple studies have shown that GAS6 is secreted by diverse cell types, from the tumor and/or stromal cells. To cite a few examples, autocrine activation and production of GAS6 by tumor cells have been described for melanoma, GIST, and breast cancers [39–42]. Secretion of GAS6 from the tumor microenvironment has been shown in colon, breast, and prostate cancers as well as in AML. In glioblastoma, breast cancer, and AML, both autocrine and paracrine secretion of ligands have been detected [6, 43]. The production of GAS6 by stromal cells can create a specific niche in which AXL signaling cascades are activated and favor metastasis development [44••]. Apart from ligand binding, little is known as to the regulation of AXL/MER activation. A soluble form of AXL/MER has been described to negatively regulate AXL/MER signaling by acting as an antagonist to GAS6 [45, 46]. The C1 domain-containing phosphatase and tensin homolog protein (C1-TEN) can dephosphorylate AXL and block downstream AKT activation [47]. AXL protein can be stabilized by binding to heat-shock protein 90 (HSP90) [48] or destabilized by ubiquitination by the casitas B-lineage lymphoma (CBL) E3 ligases [49, 50]. Interestingly, a downregulation of CBL has been described as playing a pivotal role in the resistance of CML to BCR-ABL inhibition [51].

### AXL and MER Downstream Signaling Pathways

Like most RTKs, AXL and MER transmit signals into the cell via adaptor proteins. Importantly, the binding of adaptor proteins to phosphorylated AXL/MER appears to be context- and tissue-specific. It is thus important to understand the disease-specific phenotype in order to decipher in which signaling networks AXL and MER play a role. Activation of AXL/MER by ligand binding triggers receptor dimerization and subsequent autophosphorylation of their cytoplasmic domain [1••]. Each phosphorylated tyrosine residue (Tyr) serves as a docking site for specific adaptor proteins. Five phosphorylation sites have been described for MER: Tyr749, Tyr753, Tyr754, Tyr872, and Tyr929. The last two mediate binding to GRB2 and the p85 regulatory subunit of PI3K, which activate MEK/ERK and PI3K/AKT signaling pathways, respectively. Six phosphorylation sites have been identified in AXL: Tyr698, Tyr702, Tyr703, Tyr779, Tyr821, and Tyr866. While the three more N-terminal residues are putative autophosphorylation sites reflecting AXL activation, the three most C-terminal tyrosines are docking sites for downstream effector proteins [52]. Phospho-Tyr779 and phospho-Tyr821 bind to p85 and activate PI3K/AKT signaling; binding of GRB2 to phospho-Tyr821 transduces the MEK/ERK cascade; phospho-Tyr821 and phospho-Tyr866 bind to SRC, LYK, and PLCγ which can switch on additional signaling networks such as PKC or STAT [53–56] (Fig. 1).

### Direct Roles of AXL and MER Signaling in Resistance

AXL expression can circumvent resistance to targeted agents and specifically to inhibitors of other RTKs by either maintaining activity of the same pathway via alternative effectors or by inducing activation of distinct signaling networks. For instance, in NSCLC and HNSCC models, AXL expression sustains PI3K/AKT and MEK/ERK signaling and thus mediates resistance to the EGFR inhibition [24, 57]. A positive feedback loop further reinforces this bypass mechanism in which the MEK/ERK pathway induces transcription of *AXL* by JUN [24]. Moreover, AXL can dimerize with non-TAM receptors, such as EGFR, MET, and PDGFR [25, 58••, 59, 60, 61••]. This crosstalk triggers a signaling switch, leading to the bypass of the RTK inhibitor effect. An elegant study in triple-negative breast cancers demonstrated that AXL binds to EGFR, other ErbB receptors, MET, and PDGFR depending on their expression levels and membrane localization [58••]. Similarly, dimerization of AXL and HER3 has been shown to bypass HER2 signaling inhibition by lapatinib in breast cancer cells [35]. In squamous cell carcinoma resistant to PI3K inhibitors, dimerization of AXL with EGFR activates the PLCγ/PKC/mTOR pathway to sustain tumor progression [61••], and in mesenchymal ovarian tumors and cell lines, AXL dimerized with MET, EGFR, and HER2, leading to sustained ERK activation [62•]. Switching between receptors is another escape mechanism that has been described in gastro-intestinal stromal tumors (GIST). Cells resistant to the KIT/PDGFR inhibitor imatinib express lower levels of KIT while upregulating AXL and GAS6 [39]. Finally, a recent study has very elegantly shown that inhibition of the MEK/ERK pathway decreases the proteolytic shedding of AXL from the surface of melanoma and breast cancer cells, thus removing negative feedback on its signaling activity [63•].

### Indirect Roles of AXL and MER in Resistance via Tumor Cell Proliferation, Survival, Migration, and Invasion

As described above, activation of AXL or MER initiates signaling cascades that are essential for tumor progression. While regulation of cell proliferation is mostly mediated by activation of the p38- and MEK-driven MAPK signaling cascades, most of the published work suggests a more prominent role of AXL in the PI3K/AKT/mTOR and JAK/STAT pathways in tumor cell survival [1••, 2, 10, 64] (Fig. 1). In particular, activation of AKT leads to the nuclear translocation of NF-kB which induces expression of anti-apoptotic proteins, such as survivin, BCL2, BCL-XL, and cyclinD1, as well as phosphorylation and inhibition of the pro-apoptotic protein BAD [10, 65–67]. As a consequence, inhibition of AXL/MER can induce apoptosis in tumor cells via blocking both MEK/ERK and PI3K/AKT pathways [68]. One study in chronic lymphocytic leukemia (CLL) also suggested that AXL inhibition mediates apoptosis by reducing the expression of the anti-apoptotic protein MCL1 [54]. Induction of the PLCγ-PKC signaling cascade by AXL leads to mTORC1 activation, which also promotes cell survival [61••] (Fig. 1). In addition, interplay between receptors of the TAM family can modify the signaling outcome induced by GAS6 [69]. Interestingly, one study suggests that the balance between AXL and TYRO-3 expression is important in the dormancy of prostate cancer cells, with high TYRO-3 levels promoting proliferation and high AXL expression leading to a quiescent phenotype [70].

Besides regulating proliferation and survival, AXL promotes cell migration, cell invasion, and metastasis development in several cancer types [7, 8, 71–73]. The induction of such a migratory phenotype is mediated by AKT and SRC pathways as well as RAC-induced cytoskeleton changes [74, 75]. It was also proposed that the kinase domain of AXL can bind to the actin cytoskeleton while the extracellular domain of the receptor modulates cell adhesion by regulating the expression of tight- and adherent-junction proteins [76]. Together, these observations strongly suggest that the increase of AXL/MER activity typically observed following conventional or targeted therapy will lead indirectly to drug resistance via marked promotion of tumor progression and aggressiveness (Table 1).

## AXL and MER in Resistance Mediated by Epithelial-to-Mesenchymal Transition

### The Importance of EMT in Resistance

Epithelial-to-mesenchymal transition, or EMT, corresponds to the reversible conversion of epithelial cells to mesenchymal cells, and plays an important role during embryonic development and wound healing. EMT involves profound phenotypic changes that include loss of epithelial characteristics with concomitant acquisition of mesenchymal traits, the latter being more appropriate for migration and invasion. In the cells that undergo EMT, typical epithelial markers, such as E-cadherin and cytokeratins, are repressed while mesenchymal markers, such as N-cadherin, vimentin, or fibronectin, are induced. A number of transcription factors are well described as EMT inducers: SNAI1/2, TWIST1/2, and ZEB1/2. EMT is also associated with modifications in matrix composition and matrix adhesion proteins that will contribute to enhance cell migration in the stromal compartment [77]. Due the plasticity of cells required during the invasion cascade, multiple studies have shown an association between EMT and metastasis development [78].

The loss of epithelial features by mesenchymal cells is very often correlated with induction of stem cell-like properties, such as decreased proliferation and as a consequence, increased resistance to anti-proliferative agents [79]. In this way, cells that are intrinsically mesenchymal or that have undergone an EMT show higher degree of resistance to chemotherapeutic agents as well as to targeted therapies [80•]. Interestingly, a recent study in mesenchymal NSCLC, TNBC, and HNSCC cell lines very elegantly demonstrated that the reversal of EMT by AXL inhibition was accompanied by decreased expression of DNA repair genes, diminished efficiency of homologous recombination and sensitivity to poly (ADP-ribose) polymerase (PARP) inhibition, leading to apoptotic cell death [81••].Together, these studies show that EMT can be considered a major, albeit indirect, mechanism of drug resistance [82•].

### AXL as a Sustainer and Effector of EMT

The implication of AXL in EMT is supported by a multitude of studies, which frequently highlighted a correlation between AXL expression and features of EMT [77, 80•, 83]. Expression of AXL is upregulated in mesenchymal EMT-like cells, suggesting a role of AXL in this phenotypic transition. In addition, *AXL* scores as one of the top genes in EMT-specific signatures [44••, 82•, 84•]. AXL expression enhances migratory capabilities of cancer cells and is often associated with increased metastasis development and poor prognosis. However, whether AXL induces EMT or whether EMT induces AXL expression remains an open debate. More mechanistic data on their causal relationship are warranted to address this point.

Inhibition of AXL by small molecule inhibitors or depletion of AXL by siRNA has been shown to reverse resistance of mesenchymal cancer cells, without necessarily switching them back to an epithelial state. These findings support the fact that AXL is required to maintain EMT-driven drug resistance, but is not necessarily the cause of the mesenchymal state itself [10, 44••,^85^]. Indeed, several reports pinpoint AXL as a downstream effector of EMT. Most available data are focused on breast cancer, where SNAI1/2 transcription factors induce AXL expression together with regulation of the expression of key EMT genes [42, 86]. An EMT gene signature, which includes high levels of *AXL*, has been described as a predictive biomarker of resistance to EGFR or PI3K inhibitors in several solid tumors. However, in this context, inhibition of AXL by a small molecule inhibitor was sufficient to reverse EMT-associated resistance [82•]**.** Supporting this finding, a kinome-wide shRNA screen also identified *AXL* as a key regulator of the mesenchymal state and stem cell properties in glioblastoma [87]. A similar study in breast cancer models demonstrated a role of AXL in the maintenance of stemness and further showed that AXL downregulation could reverse the EMT phenotype of the cancer stem cell population [88]. A number of mechanistic studies also support the hypothesis that AXL is indeed an EMT inducer. AXL was shown to control the expression of the transcription factors SNAI1/2 and TWIST1/2 in pancreatic cancer [89] and, in breast cancer, to activate the AKT/GSK3β/β-Catenin cascade that induces expression of ZEB1 and other EMT-related genes [90]. A similar study performed in head and neck cancer suggests that resistance to the EGFR inhibitor erlotinib is associated with low miR34a and high AXL levels, the latter inducing EMT via the AKT pathway [28].

In conclusion, the causal connection between AXL and EMT is likely to be context- and tissue-specific. Since several reports have clearly highlighted the therapeutic value of inhibiting AXL/MER signaling cascades, AXL/MER receptors are likely not solely biomarkers of the mesenchymal phenotype and drug resistance but rather have a functional role in maintaining this drug tolerant state.

## AXL and MER in Resistance Mediated by the Tumor Microenvironment

### Role of AXL and MER in the Innate Immune Response

Besides the oncogenic signaling networks described in the previous sections, AXL and MER play important roles in the innate immune system by promoting phagocytosis of apoptotic cells and debris, supporting the maturation of natural killer cells (NK cells) and inhibiting inflammation driven by dendritic cells (DCs) and macrophages [1, 91, 92•]. Here, the functions of AXL and MER as regulators of inflammation are discussed in the context of their role in the immune response to cancer and resistance to anticancer treatment.

In inflammatory conditions, type I interferon (IFN) binds the IFNα receptors (IFNAR) on DCs to amplify the inflammatory response through activation of the JAK/STAT1 pathway, leading to the transcription of pro-inflammatory cytokines. AXL and MER have been shown to bind the IFNAR and redirect downstream signaling via STAT1 to activate transcription of the suppressor of cytokine signaling (SOCS) proteins which inhibit JAK [92•]. Thus, AXL/MER activation mediates a negative feedback loop in order to dampen the inflammation process and avoid tissue damage. Furthermore, activation of the TAM receptors in macrophages leads to a switch from a M1 to a M2 phenotype, the latter being unable to activate CD8 positive T cells [93]. As a consequence, activated TAM receptors in tumors induce immunosuppression which blocks the antitumor activity of cytotoxic T cells. In such an environment, the efficacy of anticancer treatment is decreased and resistance can develop. Supporting this hypothesis, a high level of M2 macrophages in tumors often correlates with poor prognosis. On contrary, inhibition of TAM receptors maintains macrophages in a M1 state, in which they secrete pro-inflammatory cytokines and can activate T cells [1••, 94, 95•].

Taking this role of AXL/MER into account, blocking their activity could improve antitumor response by (i) increasing pro-inflammatory cytokines and antitumor activity of cytotoxic T cells in tumors with an immunosuppressive environment (high M2 macrophages, low levels of activated CD8 T cells), (ii) simultaneously targeting tumor cells and macrophages in resistant AXL/MER-positive tumors, and (iii) combining TAM inhibitors with immunotherapies to improve antitumor T cell activity by blocking inhibitory checkpoints (e.g., anti-PD1 or anti-CTL4A). In support of this approach, a recent study by Hugo et al. in metastatic melanoma demonstrated that innate resistance to anti-PD1 therapies was associated with overexpression of AXL and an increased number of infiltrated macrophages [96••]. Of note, three recent studies in mouse syngenic models of colon and breast cancer showed marked synergy for tumor growth inhibition with dual inhibition of AXL and PD1 or CTLA4 [97•, 98•, 99•]. Incidentally, a combination of AXL/MER inhibition with immunotherapies may be of particular importance in tissues where inflammation could have a tumor-promoting function, as is the case of colon cancer [100].

In conclusion, these findings suggest that AXL/MER inhibitors could have an important tumor immunomodulatory role, causing a switch from an anti-inflammatory and immunosuppressive context (M2 macrophages) to a pro-inflammatory and immuno-active milieu (M1 macrophages, activated T cells). Immunoprofiling of patient tumors pre- and post-treatment and its correlation with TAM expression as well as more detailed preclinical studies in immunocompetent models are required to validate this hypothesis.

### Role of AXL and MER in Angiogenesis

Another function of TAM receptors resides in vascular integrity and pro-angiogenic properties. During wound healing or vasculature damage, TAM receptor signaling promotes stabilization of platelet aggregation, survival of endothelial cells, and restitution of the endothelial barrier function [92•, 101–103]. Being expressed by endothelial cells, TAM receptors participate in the formation of new vessels and contribute to their stabilization via signaling in AXL-positive vascular smooth muscle cells. In particular, AXL has been described as a key modulator of endothelial cell functions that are required for angiogenesis and tumor growth [104]. Both autocrine and paracrine loops between AXL and its ligands GAS6 promote motility and proliferation of endothelial cells, modulate integrin function to facilitate migration and survival of endothelial and tumor cells, and facilitate cell motility via regulation of RAC and AKT pathways. In this context, activation of AXL and MER on endothelial cells has been proposed as a means of resistance to antiangiogenic therapies targeting the vascular endothelial growth factor (VEGF) and fibroblast growth factor (FGF) receptors [102, 103, 105]. A recent phase III clinical study of cabozantinib—which inhibits AXL in addition to MET and VEGFR—in renal cell carcinoma (RCC) patients progressing after VEGFR inhibitor treatment, supports the hypothesis that AXL inhibition could target resistance to VEGFR inhibition [106].

## Targeting AXL and MER in the Clinic

### Optimal Therapeutic Strategies as a Function of Cancer Type and Resistance Mode

Overall, based on the current evidence, it seems that AXL and MER have a limited role to play in cancer initiation and progression per se. AXL/MER overexpression seems to be largely restricted to cells that are or have become refractory to anticancer treatments, where their roles in cancer cell proliferation, survival, migration, invasion, and EMT provide a strong rationale to block their activation in order to reverse the drug tolerant state and overcome resistance. AXL/MER inhibitors could thus be used in two distinct scenarios, as modulators of either innate or acquired resistance.

Some cancers have intrinsically high AXL or MER expression. Studies suggesting a role of AXL and MER in leukemia cells led to a phase I clinical trial of the specific AXL inhibitor BGB324 as a monotherapy in AML in which signs of clinical benefit were seen [107••]. Studies on treatment-naive colon and breast cancers have described mesenchymal subgroups with high AXL expression, suggesting that targeting of AXL in these specific patient populations could be beneficial, possibly as a monotherapy or, more likely, to reverse innate resistance to conventional and targeted therapies [108•, 109, 110]. However, a wider benefit of AXL/MER inhibitors is likely to be for patients with acquired resistance to conventional or targeted therapies, where AXL/MER inhibitors would be used in combination with the agent to which resistance was developed. Preclinical and clinical studies that form the basis of the different rationales for these combinations are summarized in Table 1; some are described in more detail in the preceding sections. Several clinical trials are currently evaluating the potential of AXL/MER inhibitors in these settings [10, 111•, 112]. Finally, the immunosuppressive and pro-angiogenic functions of TAM receptors place them as attractive targets for the tumor stroma. Indeed, inhibition of TAM could create an immune proficient niche and favor activation of cytotoxic T cells. Blocking AXL signaling could also reduce angiogenesis and tumor growth [105]. Hence, AXL/MER inhibitors could have a dual function by targeting both tumor cells and their stroma. However, careful toxicity evaluation should be done as chronic exposure to TAM inhibitors, particularly MER, may cause autoimmune disorders. There is, nonetheless, a certain degree of redundancy in the roles of the TAM receptors in immune suppression. The development of specific AXL inhibitors with lower affinity for either MER or TYRO-3 could thus be useful to avoid these potential side effects [1••, 92•].

### Small Molecule AXL and MER Inhibitors in Preclinical and Clinical Development

Three strategies have been developed to inhibit TAM activity by (i) blocking ligand/receptor binding with antibodies, (ii) inhibiting kinase activity with ATP competitors, and (iii) reducing TAM expression [111•]. Anti-AXL monoclonal antibodies and an aptamer approach have been described but these two strategies have not been well documented so far [105, 113–116]. The second strategy, which is commonly used for RTK inhibition, has led to several ATP-competitive compounds [10, 111•, 112].

Many of these molecules are multi-kinase inhibitors, for which AXL is not the main target and that were not initially developed to block its activity (Table 2). So far, only three molecules are described as specific AXL inhibitors: the first-in-class compound BGB324 which has entered clinical trials and TP-0903 and SLC-0211 still at preclinical stage. The lack of specificity of some of the other molecules may nevertheless be used as an advantage for anticancer treatment as multiple RTKs are involved in tumor progression and disease recurrence. Due to the similarity of the ATP binding site between MET and AXL, many of the compounds target these two receptors. As MET and AXL are involved in resistance mechanisms, especially in NSCLC, inhibiting both simultaneously may target distinct resistant populations within the same tumor, or prevent the emergence of secondary mechanisms of resistance, and thus be highly beneficial for patients. In this context, monotherapy trials of the multi-kinase inhibitors Cabozantinib, Sitravatinib, and Glesatinib are specifically including NSCLC patients with high expression or genetic aberrations of AXL. However, based on the preclinical hypotheses outlined in this review, it may be necessary to combine with the agent to which resistance has developed to observe clinical benefit. Moreover, the fact that these multi-kinase inhibitors also target angiogenesis via inhibition of VEGFR2 will further confound the issue. The interpretation of the results of these trials will thus have to be made with caution in terms of any possible link between antitumor efficacy and high AXL expression. Results of the phase I/II NSCLC trials of the more selective Gilteritinib and BGB324 (no specific patient selection) and S49076 (including patient selection based on high AXL expression) in combination with EGFR inhibition are eagerly awaited. BGB324 is also being investigated in combination with docetaxel in NSCLC.

**Table 2:** AXL and MER inhibitors in preclinical and clinical development

Drug other names; developer	Targets	Indications	Phase
Cabozantinib XL184, Cometriq®; Exelixis/Ipsen	VEGFR2, MET, RET, AXL	Thyroid NSCLC^a^, RCC, other solid tumors	MA II/III
Bosutinib SKI-606; Bosulif®; Pfizer	BCR-ABL, SRC, AXL	CML breast, GBM, other solid tumors	MA II
Glesatinib MGCD265; Mirati	MET, VEGFR2, RON, AXL	NSCLC^a^, bladder, breast	II
Merestinib LY2801653; Eli Lilly	RON, MET, AXL, FTL-3	NSCLC, biliary tract	II
Gilteritinib ASP2215; Astellas Pharma	AXL, FLT3	NSCLC^a^ (+EGFRi), AML	II
BMS-777607 ASLAN002; BMS/Aslan	AXL, RON, MET, TYRO3, FLT3	Solid tumors	I/II
S49076 Servier	MET, MER, AXL, FGFR1–3	GBM (+VEGFi) NSCLC^a^ (+EGFRi)	I/II
BGB324 R428; BerGenBio	AXL	AML, NSCLC (+EGFRi)	I/II
Sitravatinib MGCD516, Mirati	VEGFR2, PDGFRA, AXL, MER, RET, MET,	NSCLC^a^, other solid tumors	I
	DDR2, TRKA		
Ningetinib HEC Pharm	VEGFR2, MET, AXL, MER, FLT3, RON	GBM, other solid tumors	I
BPI-9016 M Betta Pharmaceuticals	MET, AXL	Solid tumors	I
TP-0903 Tolero Pharmaceuticals	AXL	–	Preclinical
ONO-9330547 Ono Pharmaceutical	AXL, MER	–	Preclinical
SLC-0211 SignalChem Lifesciences	AXL	–	Preclinical
NPS-1034 NeoPharm	AXL, FLT3, KIT, MET, ROS1, TIE1	–	Preclinical
LDC1267 Lead Discovery Center	MER, TYRO3, AXL	–	Preclinical
UNC2250 University of N. Carolina	MER	–	Preclinical
UNC2025 University of N. Carolina	MER, FLT3, AXL, TYRO3	–	Preclinical
RXDX106 Ignyta	AXL, MER, TYRO3, MET	–	Preclinical

Sources of information on clinical trials include ClinicalTrials.Gov (https://clinicaltrials.gov/) and TrialTrove (https://citeline.com/). Targets are listed in order of potency (most potently hit first) based on available information

*MA* market authorisation

^a^Patients selected based on high expression or genetic aberrations of AXL

## Challenges in Identifying Pharmacodynamic and Predictive Biomarkers

The signaling cascades activated downstream of AXL/MER are dependent on the tissue context. These complex signaling networks have been a challenge for the identification of robust biomarkers of (i) AXL/MER activity and (ii) response to AXL/MER inhibition. Moreover, the discrepancy between the possible readouts proposed in different in vitro studies is, in part, due to the lack of robust relevant in vitro models to study AXL/MER cellular functions. Indeed, their activity is tightly regulated by interaction with their ligands, which may require the presence of stromal cells and/or a particular organization of tumor cells. More complex 3D co-culture systems may resolve this issue. So far, molecular data are mostly available on AXL rather than MER as it is the most commonly studied and targeted TAM receptor.

### Pharmacodynamic Biomarkers

Levels of expression and activation of AXL in patient tumors are currently assessed by immunohistochemistry analysis of total protein and phosphorylation status of the receptors as well as expression of GAS6. The phosphorylation of the Tyr702 site, for which good antibodies are available, is commonly used as a marker of AXL activation.

The identification of a robust pharmacodynamic (PD) marker for direct target hitting has been challenging, largely due to the fact that there are no good commercially available antibodies to the autophosphorylation sites (Tyr779, Tyr821, and Tyr866). Interestingly, while some of the less selective AXL inhibitors may also indirectly lead to a marked reduction in phosphorylation of the Tyr702 site [117], more selective inhibitors such as BGB324 and S49076 do not [118, 119]. The implications of these different patterns of inhibition of the phosphosites in terms of downstream signaling remain to be determined. Good and specific antibodies for each docking site are also lacking, which precludes a precise molecular analysis of AXL phosphorylation status.

The activation of multiple signaling cascades, together with the fact that many if not all of these pathways are also downstream of other RTKs, excludes the use of phosphorylation of downstream molecules such as AKT. It is possible, however, that further insight into the impact of AXL-specific compounds on signaling via in depth phospho-proteomic studies would provide useful, specific, PD biomarkers.

### Predictive Biomarkers of Response

The expression levels of AXL, and to a lesser extent GAS6, are described as broad markers of poor prognosis [6, 120]. In AXL-driven clinical trials, AXL and GAS6 expression levels are used to select AXL-positive populations. What threshold of AXL/GAS6 expression or whether a specific molecular context is associated with a better clinical response is still an open question. The comparison of responders and non-responders from the ongoing BGB324 and S49076 clinical trials will likely be very informative.

## Conclusion

Resistance to conventional and targeted therapy is a major cause of failure of anticancer treatment. Further understanding of resistance mechanisms and identification of specific targets driving this resistance will allow development of compounds able to selectively kill the drug tolerant population and avoid disease recurrence. TAM receptors, in particular AXL, have emerged as key mediators of innate and acquired drug resistance in multiple cancer types, from both hematological and epithelial origins. As a consequence, several multi-kinase inhibitors have been repurposed to target AXL in the clinic to reverse resistance. Importantly, the role of AXL in dampening the immune response has led to promising novel therapeutic strategies combining AXL targeting compounds with immune checkpoint inhibitors. In conclusion, AXL has become an attractive target for anticancer treatment, most specifically in combination. More AXL-specific inhibitors are now being developed and will hopefully provide novel strategies to overcome drug resistance using well-tolerated drugs capable of being used in combination. The identification of robust biomarkers of activity and response for these novel molecules will be required for appropriate patient stratification and optimal clinical benefit.
